# Study on the battery thermal management system for cylindrical lithium-ion battery with nano-doped phase change material and liquid cooling

**DOI:** 10.1038/s41598-025-08884-5

**Published:** 2025-07-05

**Authors:** Dhinesh Balasubramanian, Inbanaathan Papla Venugopal, Mohankumar Subramanian, Vijayanandh Raja, Utku Kale, Jonas Matijošius

**Affiliations:** 1 Department of Mechanical Engineering, Mepco Schlenk Engineering College, Sivakasi, Tamil Nadu India; 2 Department of Automobile Engineering, Kumaraguru College of Technology, Coimbatore, Tamil Nadu India; 3 Department of Aeronautical Engineering, Kumaraguru College of Technology, Coimbatore, Tamil Nadu India; 4 Department of Aeronautics and Naval Architecture, Faculty of Transportation Engineering and Vehicle Engineering,, Budapest University of Technology and Economics, Műegyetem rkp. 3., H-1111 , Budapest, Hungary; 5 Mechanical Science Institute, Vilnius Gediminas Technical University, Plytinės g. 25, LT-10105 Vilnius, Lithuania; 6 Department of Port Engineering, Lithuanian Maritime Academy (LMA) Vilnius Gediminas Technical University , Klaipėda,, Lithuania

**Keywords:** Battery thermal management system (BTMS), Hybrid cooling, Phase change material (PCM), Energy science and technology, Engineering, Materials science

## Abstract

The rapid rise in global warming pushes the automobile industry towards adopting Electric vehicles globally. The battery is considered a core element of electric cars and must operate in extreme conditions. Therefore, a suitable heat recovery system must be implemented to make the battery reach its higher performance. This work explores and tests a hybrid-based Battery Thermal Management System under various operating conditions. To enhance the property of paraffin (Phase Change Material) in the passive method, Al_2_O_3_ nanoparticles have been added with paraffin in three different ratios of 5%, 10%, and 15%. In the case of the active method, the water is circulated in a counterflow direction through the copper coil. A detailed experimental investigation was carried out at free convection, pure paraffin, PCM with Al_2_O_3_ (5%, 10%, and 15%), and hybrid (both active and passive). Each investigation was carried out under different C rates (0.5 C, 1 C, 2 C, 3 C). The result shows that under natural convection for a 3 C discharge rate, the temperature rises to 51.16 °C, and it is reduced to 40.81 °C for hybrid conditions. Finally, after incorporating the hybrid-based thermal management system, a temperature reduction of around 10.35 °C is observed when compared with natural convection conditions. A detailed computational study was also carried out to validate the experimental results. The maximum temperature of 47 °C is obtained for a pure PCM battery module at a 3 C discharge rate, and it is well matched with experimental results.

## Introduction

In recent days, the shortage of non-renewable fuels and carbon emissions has created a major problem worldwide. Consequently, the entire world is moving toward a system that provides high energy without any carbon emissions. This can be achieved only by employing electric vehicles. The Li-ion battery plays a crucial role in Electric Vehicles (EVs) due to its longer life cycle, high energy density, and low self-discharge rate^1–3^. During the anode-cathode reaction of the cell, the temperature of the battery continues to rise. If the generated heat in the battery is not effectively dissipated to the surroundings, the battery begins to degrade. The operating range of lithium-ion batteries is 25–40 °C, and the temperature difference between the cells in the battery pack should be less than 5 °C^4–6^. If the temperature of the batteries in the pack exceeds this working range, it can lead to thermal runaway and may even result in the fire and explosion of the battery packs. The discrepancy between the State of Charge (SOC) and localized degradation is attributed to this thermal non-uniformity^7,8^. To maintain the battery’s temperature within the intended working range, an effective battery thermal management system must be developed.

Active, passive, and hybrid battery thermal management systems (BTMS) are commonly available types of BTMS. The active approach is more dependable and adjustable since it uses an external power source to lower battery temperature. Some of the active cooling methods in BTMS include forced air cooling, liquid cooling with circulating water or another coolant, and refrigeration cycle cooling^9–11^. In a passive BTMS, the battery temperature can be reduced without any external energy consumption, making it a more energy-efficient thermal management process. Common passive methods for managing temperature in the battery pack include phase change materials (PCM) and heat pipes that transfer heat within the battery pack^12^. However, there are some problems with PCM-based BTMS; for example, paraffin-based PCM has lower thermal conductivity^50^, leading to poor performance in battery thermal management^13,14^. Adding materials with excellent heat conductivity, such as expanded graphite, can help solve these issues. Graphite^15,16^, carbon fibre^17,18^, metal foam^19,20^, and nanoparticles like Al2O3 and CuO^21^ can also serve as effective solutions. Recent research is accelerating toward hybrid BTMS, which combines both active and passive systems to utilize the benefits of both systems^22,23^. Peng Qin et al. (2019) conducted an experiment and numerical analysis using PCM with air-forced convection to obtain a hybrid-based BTMS. Results obtained revealed that in natural convection conditions at 1 C rating and 2 C rating, the value for maximum temperature is 45.20 and 70.20, respectively. After implementing forced air convection temperature of the battery is further reduced to 10 °C^24^. In a similar study, they analysed the thermal performance of seven cases, which include two cases with no TMS (thermal management System), two active TMSs, one passive TMS, and two hybrid TMSs. Polypropylene glycol 1000 is used as a PCM, and a detailed analysis was carried out. The result conveys that from all cases. The seventh case shows that the maximum temperature declined from 58 °C to 32 °C^25^. Depeng Konga et al. (2020) proposed a coupled composite PCM with a liquid cooling thermal management system for the continuous operation of a lithium-ion battery pack under different ambient temperatures. According to test data, after a 3 C discharge, the battery pack’s maximum surface temperature and temperature differential were 41.1 °C and 4 °C, respectively^26^.

To control the temperature of Li- ion batteries, Qiqiu Huang et al. (2021) conducted an experiment using a unique flexible form-stable composite phase change material. The results show that at a ratio of 2:1, the composite PCM exhibits high impact resistance. Under composite PCM conditions for a 5 C discharge rate, the maximum battery temperature reaches 46 °C, with a temperature difference of 4 °C^27^. Another study included a thorough experimental analysis of a composite phase change material made from expanded graphite and paraffin to manage the thermal state of lithium-ion batteries. According to the results, the battery temperature drops from 77 °C to 43 °C under 3 C discharging conditions. This composite PCM enables the battery temperature to be maintained between 20 °C and 55 °C across all other discharge rate settings^28^. Raja Elarem et al. carried out an experimental analysis to identify the variation in latent heat, viscosity, and melting temperature for different sizes, shapes, and concentrations of nanoparticles. In this investigation, various mass fractions of copper (Cu) and aluminium (Al) were added to paraffin wax to create nano-enhanced phase change materials (1%, 2.5%, 5%, 5%, etc.). Regarding the thermal storage properties of paraffin, the 2% and 1% mass fractions of Al and Cu nanocomposites perform better than the higher mass fractions. In recent decades, several studies have explored various layouts of battery modules. Among the tested battery modules, hexagonal-based designs provide better heat transfer compared to others^29^. Yijie Zhuang et al. conducted a numerical analysis of a hybrid BTMS, where PCM and cooling plates for cylindrical Li- ion batteries are arranged in a honeycomb manner. From the analysis, they concluded that the heat dissipation effect is improved in the cooling plate with PCM, compared to without a cooling plate and BTMS^30^. The BTMS in this experiment has the following values: 32 2 A for the discharge current, 0.001 kg/s for the input flow rate, and 298.15. 15 K for the ambient temperature. The Tmax and T of the battery module are stabilized at 312 0 K and 3. 3.5 K, respectively. This technique’s proposed module prevents temperature control failure^31^. Xinyu et al. conducted a detailed simulation study for hybrid-based BTMS at various ambient temperature conditions from 20 °C to 40 °C. They simulated three conditions based on pure PCM, liquid cooling, combination of both. They have also added expanded graphite to PCM with varied cold plate thicknesses and performed simulations at different cooling rates. Test results convey that hybrid-based cooling systems perform well at a wide range of operating conditions. Regarding other parameters, 12% Expandable Graphite (EG) content with 2 mm aluminium wall thickness and a coolant velocity of 0.05 m/s performs well under all conditions^32^. In another study, they conducted pure experimental studies by incorporating EG and metal-based alloy with low melting points into pure PCM. They found that composite-based battery modules attained an elevated temperature of about 41^O^C which is lower compared with natural air convection^33^. Wang et al. attempted a numerical approach to access the performance of hybrid-based BTMS which composes of a composite fin, and cooling plate combined with PCM. Simulation results obtained convey that the designed system effectively maintains the battery operating temperature at all operating conditions^34^. Yang et al. designed a hybrid-LC (Liquid Cooling) -BTMS and compared the thermal performance with various channel counts. In this battery module, Paraffin wax RT35 was inserted as PCM into the spaces between the LCP’s liquid channels. Results obtained convey that in all operating conditions, hybrid-LC-BTMS maintains the battery module temperature below 40 °C and significantly reduces the power consumption by 50%^35^. In another study numerical approach was carried out to study the performance of hybrid-based liquid cooling BTMS. Regarding battery module design, PCM in the form of slabs is inserted between prismatic Lithium-ion cells. Test results convey that the elevated temperature of the battery module declines by 33% at a 3 C discharge rate when compared with the module without PCM^36^.

The aforementioned research works indicate that the addition of nanoparticles, the structural design of battery modules, and the implementation of hybrid cooling techniques significantly enhance the heat dissipation efficiency of battery modules. In this work, all three combinations are explored to discharge the battery more safely under extreme operating temperature conditions. The hexagonal-type battery module is selected for this research due to its superior thermal uniformity and structural compactness. To improve the properties of paraffin in the passive method, Al2O3 nanoparticles have been incorporated with paraffin in three different ratios: 5%, 10%, and 15%, to enhance its thermal conductivity and energy storage capacity. In the active method, water is circulated in a counterflow direction through the copper coil to ensure effective convective heat removal. In this study, several analyses were conducted, including free convection, PCM (only paraffin), PCM (paraffin) with Al2O3 (5%, 10%, and 15%), and hybrid (both active and passive). Each configuration was evaluated across four discharge rates (0.5 C, 1 C, 2 C, and 3 C), and this comprehensive approach is distinct from previous studies, ultimately demonstrating that the hybrid BTMS design can reduce peak battery temperatures at high C-rates, thereby ensuring safer and more reliable battery operation under extreme conditions.

## Battery module design and its considerations

### Design of battery module

**Fig. 1:**
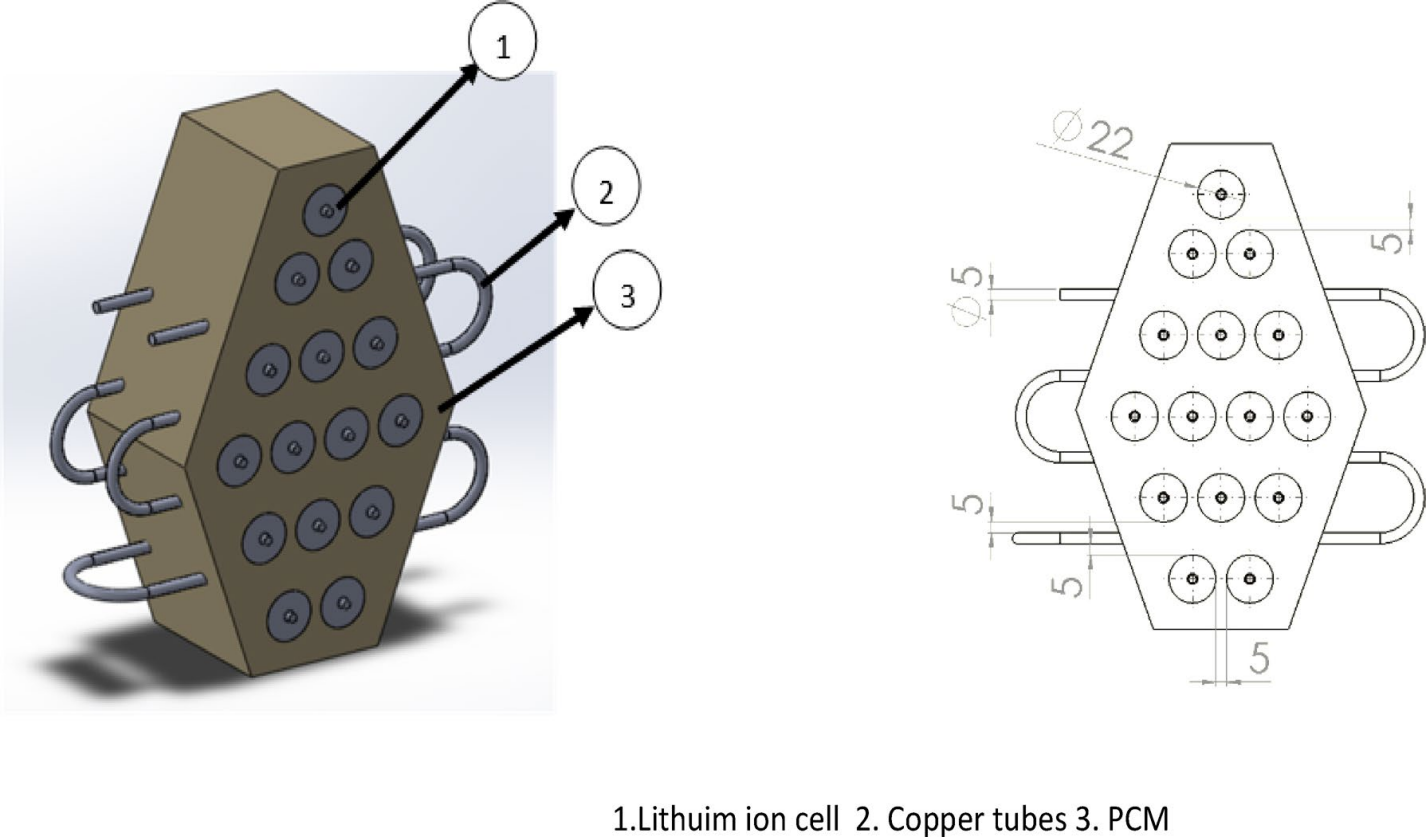
Hybrid module system design.

In E-vehicles, the main parameter to be controlled and maintained within an operating limit is temperature. By employing the merits of both active and passive methods, a hybrid technique would provide a better result than other BTMS. In this Hybrid BTMS, the Li-ion cells are embedded in the paraffin wax-enhanced Al_2_O_3_ nanoparticle setup fitted with a copper coil through which the water is circulated, as shown in Fig. 1.  2^37,38^. The paraffin wax setup is made in a hexagonal shape so that equal cell spacing is achieved. The spacing for the Hybrid setup is shown in Fig. 1. One important factor in lowering the battery pack’s maximum temperature is maintaining the distance between each cell. However, if the cell-to-cell spacing increases, which may result in a huge volume of PCM, that will create a problem with the heat dissipation for the liquid cooling system. Furthermore, the heat dissipation efficiency in the system is negligible if the cell-to-cell spacing is greater than 5 mm^26^. The cell used in this study was a cylindrical 18,650-type lithium-ion cell, which is commonly used in electric vehicles and portable electronics applications due to its high-energy density^13^. The detailed specifications of the lithium-ion battery are given in Table 1 . The properties of the paraffin wax used in this present study are given in Table 2[37,38]

**Table 1:** Specifications of Lithium-ion battery.

#	Properties	Nature
1.	Material	Li-ion battery 18650
2.	Cathode material	LiNi_0.8_Co_0.1_Mn_0.1_O_2_
3.	Anode material	Graphite
4.	Electrolyte	Solution of LiPF6
5.	Nominal capacity (Ah)	2
6.	Nominal voltage (V)	3.7

**Table 2:** Properties of paraffin wax.

#	Properties	Nature
1.	Chemical formula	C_20–40_H_42−82_
2.	Appearance	White
3.	Odor	Odorless
4.	Boiling point	370 °C (698 °F)
5.	Melting temperature	47–65 °C
6.	Heat storage capacity	56–57 °C
7.	Latent heat	250 kJ/kg
8.	Density-solid	0.88 kg/l
9.	Density-liquid	0.77 kg/l
10.	Thermal conductivity-solid	0.24 W/ (mK)
11.	Thermal conductivity-liquid	0.22 W/ (mK)
12.	Volume expansion	14%
13.	Flash point	> 200 °C
14.	Maximum operating temperature	90 °C

Similarly, the uniformity in the temperature of the battery pack could be enhanced by increasing the cell-to-plate (cell-to-coil) spacing, even though the spacing exceeds 5 mm spacing in is insignificant^39^. Concerning the detailed literature analysis, the cell-to-cell spacing and the cell-to-coil spacing of cells are 5 mm are applied in this study. The diameter of the copper tube is 5 mm. In the distribution of temperature between batteries, the effect of flow direction should be considered. The elevated heat of the battery pack in the inlet and outlet areas can be decreased by counterflow mode as compared to the parallel flow mode^39^. The flow rate of the pump, which was used for pumping the water to the tube, was 400 L/Hour (LPH).

### Preparations of the hybrid BTMS setup

**Fig. 2:**
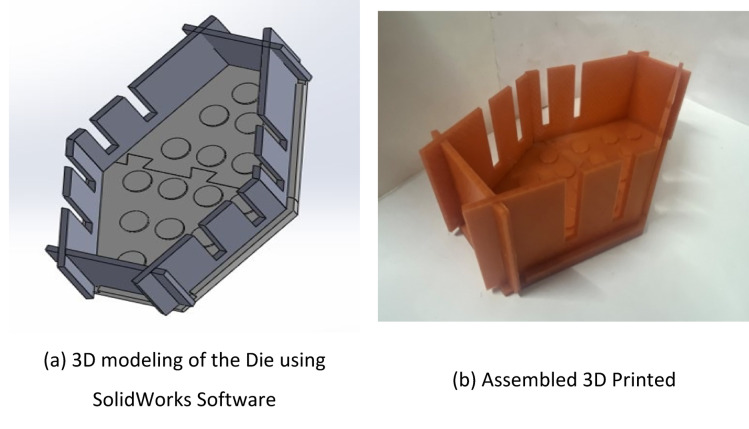
(**a**) 3D modeling of the die using solidworks software. (**b**) Assembled 3D printeddie parts.

By using SolidWorks software, the required PCM pack design was drafted as shown in Fig. 2a. The dye for preparing this hybrid model was prepared by using 3D printed technology, as shown in Fig. 2b.

The paraffin wax was melted in the oven. Within the range of 47 to 65 °C, the paraffin wax completely melted^40^, and the required percentage of Al_2_O_3_ was measured and mixed with the melted paraffin wax using a magnetic stirrer to prevent agglomeration. The mixture was then placed in an ultrasonic vibrator. The copper coil in the necessary shape was inserted into the prepared dye, after which the mixture containing paraffin with suitable percentages of 5%, 10%, and 15% Al_2_O_3_ was poured into the prepared dye and allowed to solidify for some time^21^. The nano-doped PCM and its thermal properties are presented in Table 3. After some time, the dye was removed from the hybrid pack. In this manner, the required hybrid battery thermal management pack was obtained. The entire process of preparing the Hybrid BTMS is illustrated in Fig. 3. The incorporation of Al_2_O_3_ nanoparticles into paraffin wax significantly influenced the thermophysical properties of the phase change material (PCM), as shown in Table 3.

**Table 3:** Properties of nano-doped PCM.

#	Properties	Pure PCM	PCM + 5% Al_2_O_3_	PCM + 10% Al_2_O_3_	PCM + 15% Al_2_O_3_
1.	Thermal conductivity	~ 0.2 W/m K	~ 0.4 W/m K	~ 0.62 W/m K	0.82 W/m K
2.	Latent heat of fusion	250 kJ/kg	~ 235 kJ/kg	~ 225 kJ/kg	190 kJ/kg
3.	Specific heat capacity	2.3–2.9 kJ/kg K	2.7 kJ/kg K	~ 2.5 kJ/kg K	~ 2.2 kJ/kg K
4.	Melting point	45–60 °C	62 °C	57 °C	55 °C

**Fig. 3:**
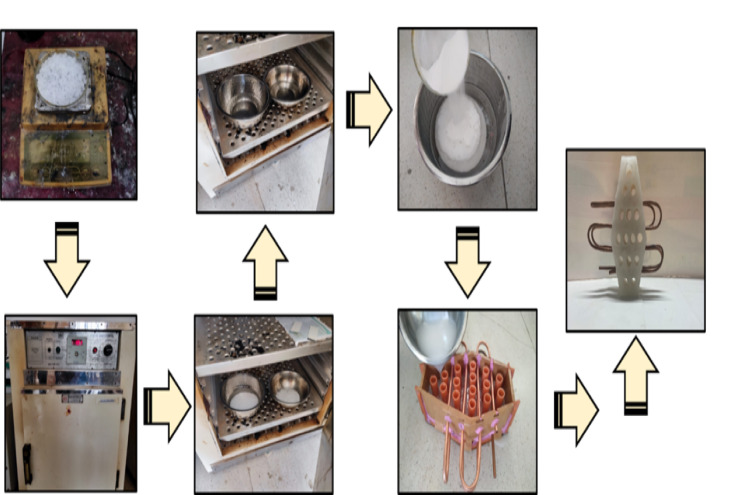
Preparation of the hybrid BTMS setup.

## Properties and chemical characterization of pcm material

### Fourier transform and infrared analysis of the test samples

**Fig. 4:**
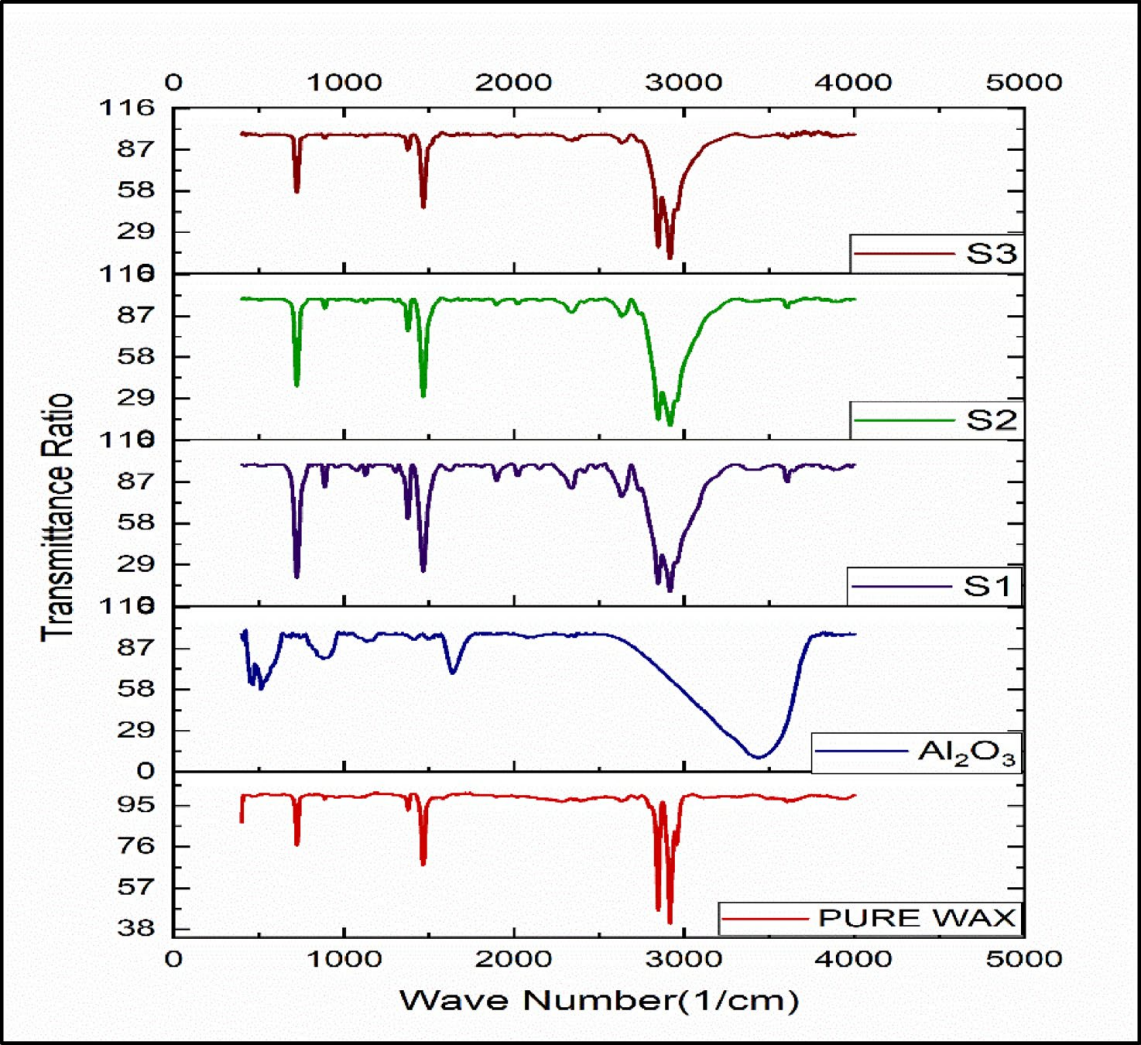
FT-IR graph of the test samples.

Fourier Transform Infrared (FTIR) spectroscopy was carried out for paraffin wax, Al_2_O_3_ nanoparticles, and paraffin enhanced with Al_2_O_3_ nanoparticles with different percentages (5%, 10%, 15%). The observed peaks in the range of 3000–4000 cm^−1^ are associated with O–H stretching vibrations, indicating the presence of hydroxyl groups, while peaks around 2850–2950 cm^−1^ correspond to C–H stretching, characteristic of alkanes in paraffin. These results confirm the identical chemical structure of paraffin wax after nanoparticle addition. Importantly, no new functional groups were observed, which implies that Al_2_O_3_ incorporation did not cause any chemical reaction or degradation of the base PCM. This ensures that the phase change behaviour of the wax remains intact, which is critical for maintaining its latent heat storage function. The peak value obtained from the test is shown in Fig. 4.

### Structure and morphological characteristics of the test samples

**Fig. 5:**
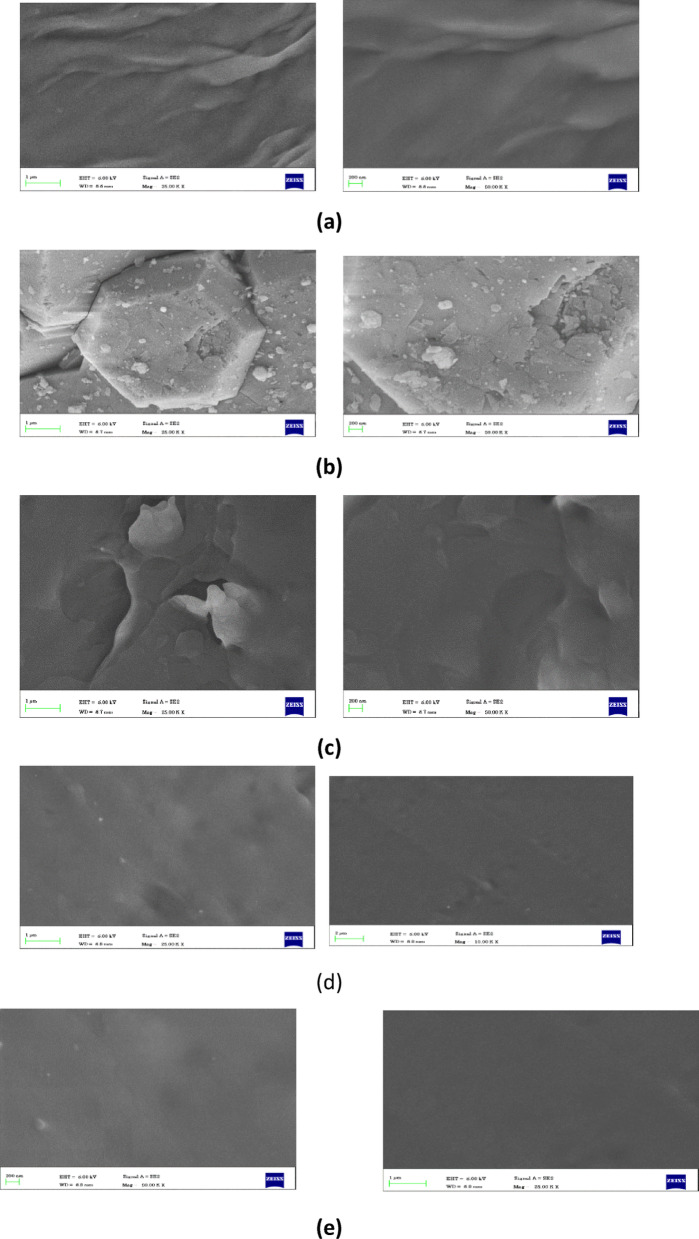
SEM image for (**a**) Paraffin Wax, (**b**) Al_2_O_3_ Nanoparticle, (**c**) Paraffin wax with 5% Al_2_O_3_, (**d**) Paraffin wax with 10% Al_2_O_3_, (**e**) Paraffin wax with 15% Al_2_O_3_.

To analyse the microstructure and morphological characteristics of the material, a scanning electron microscope (SEM) study was carried out for the material in our battery thermal management system. Figure 5a shows the SEM image of the paraffin wax under a scale of 1 μm, 200 nm. The paraffin wax has a smooth profile. Figure 5b shows the irregular shape of Al_2_O_3_ nanoparticles on a 200 nm scale. The SEM image of paraffin wax enhanced with Al2O3 nanoparticles at different percentages is shown in Fig. 5c, d, and e. At 5% and 10% Al_2_O_3_ loading, the nanoparticles are evenly distributed, forming a well-dispersed composite structure. This uniformity is crucial for consistent thermal conductivity enhancement and minimising localised hotspots during battery discharge^38^.

However, at 15% Al_2_O_3_ nanoparticle composition, over an agglomeration of particles inside the paraffin. This can cause a poor region of thermal contact, increased thermal resistance, and uneven distribution of heat over the surface. From the SEM test, we can infer that 10% Al_2_O_3_ nanoparticles have a better result with paraffin wax. It shows better morphological character, such as an even surface or even bonding with paraffin wax, compared to other results.

## Experiment test setup layout and its conditions

The charging setup for the present study includes a 12 V step-down transformer, and the 12 V AC was then converted to 12 V DC using a bridge rectifier that was linked to the transformer’s output. The 12 V DC is supplied to the cell through a breadboard. In the breadboard, a diode was inserted to avoid the backflow. From the breadboard, the supply is passed to the battery management system (BMS) to equally distribute the voltage to all cells, and the BMS is also used for the proper charging of all cells. For discharging, 12 V DC 50-watt lamps were used. Here, the cells are connected in a 3s-5p configuration (3 Series and 5 Parallel connections), and the output of this system is observed as 11.1 V, 10 Amps, which has the output value of 110 Watts. In 0.5 C rate discharging conditions, only one 12 V DC 50-Watt lamp is connected as a load for discharging. In 1 1 C rate discharging condition, two 12 V DC 50-Watt lamps were connected as a load for discharging. In 2 C rate discharging conditions, the discharging load will be twice that of the output of the battery system, and four 12 V DC 50-watt lamps were connected as a discharging load in 2 C rate discharging. In a 3 C rate discharging condition, the discharging load will be thrice that of the output of the battery system. So, six 12 V DC 50-Watt lamps were connected as a load for discharging in 3 C. Hence, the different C rating conditions were obtained for the present study. To monitor the temperature of the battery pack, an LM35 temperature sensor was utilised. LM35 is placed on the top and bottom surfaces of the individual cell, and entire connections are plugged into the Arduino setup to monitor the temperature in the laptop. The LM35 temperature sensor has low self-healing power. The output voltage range of the sensor was − 55° to + 150 °C. Its operating voltages were − 4 to 30 volts. To ensure the reliability and repeatability of the results, each experimental condition was repeated three times, and the temperature values reported are the mean of the maximum values recorded across the three trials. Temperature measurements were captured using LM35 sensors, which have a typical accuracy of ± 0.5 °C. For each test, sensors were placed at key positions as shown in Fig. 1 to monitor both axial and radial heat distribution. Across the three trials, the standard deviation of the maximum temperatures was found to be ± 0.16 °C for 0.5 °C and 1 °C, and ± 0.12 °C for 2 C and 3 C conditions. The low standard deviation values across trials with an instrument uncertainty of ± 0.5 °C and an observed standard deviation below ± 0.2 °C across sensors validate the consistency of the temperature rise behaviour under each discharge scenario.

**Fig. 6:**
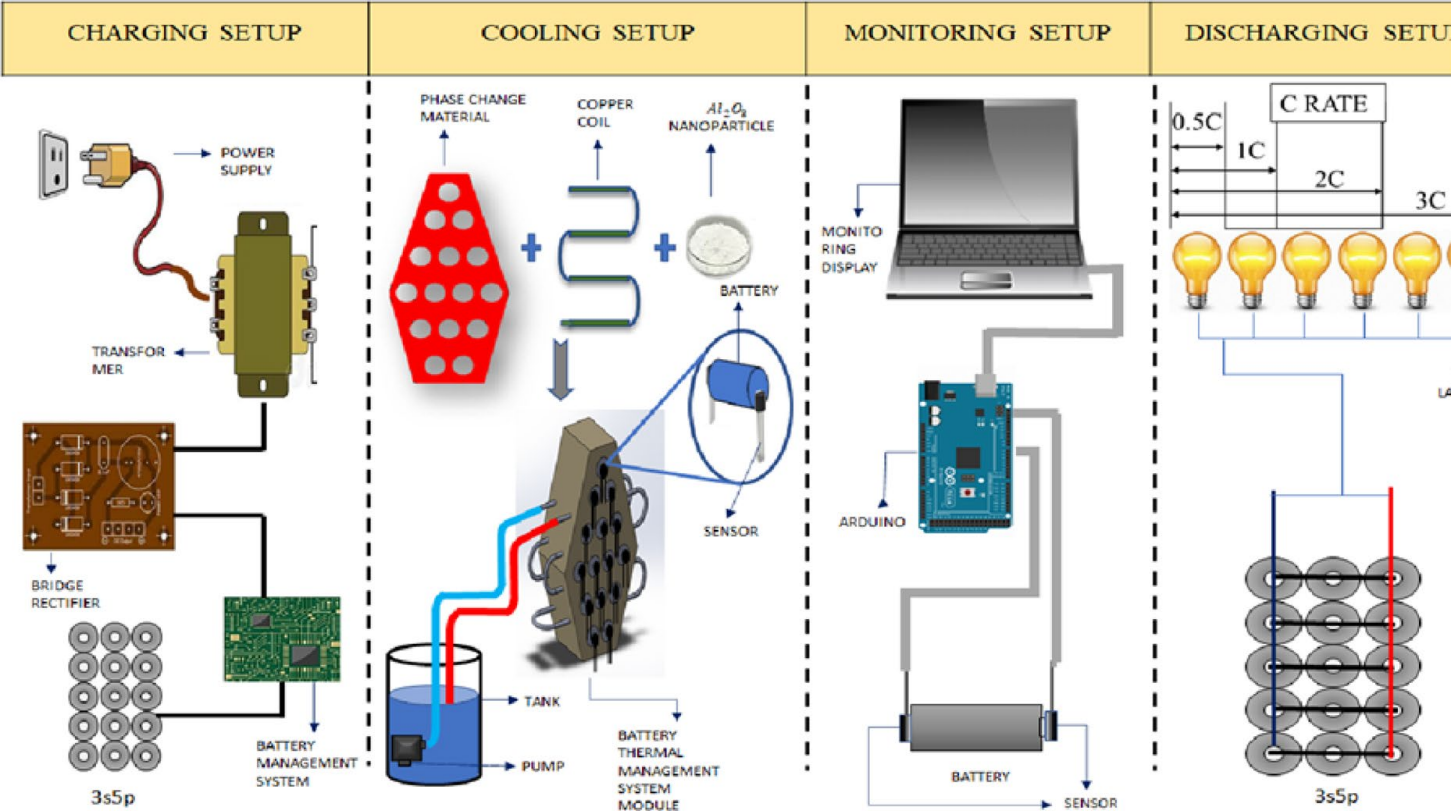
Layout of experimental test setup.

Arduino Mega 2560 was used for this experimental analysis. This Mega 2560 model can monitor 16 sensors. The ports of this Arduino are A0-A15. In this, the A0 port was used to monitor the atmospheric temperature, and the ports A1-A15 were used for 15 sensors to monitor the temperature of cells. The Arduino cable was connected to the laptop, and by inserting suitable Arduino programming into the system, we will monitor the temperature on the laptop display. The passive pack containing paraffin wax with Al_2_O_3_ nanoparticles and the copper coil to supply water through was considered an active medium, and the water was circulated in a counter-flow direction with a flow rate of 400 L/hour. The overall layout of the experimental test setup is given in Fig. 6.

## Results and discussions

### Temperature distributions for different C rates at natural convection conditions

**Fig. 7:**
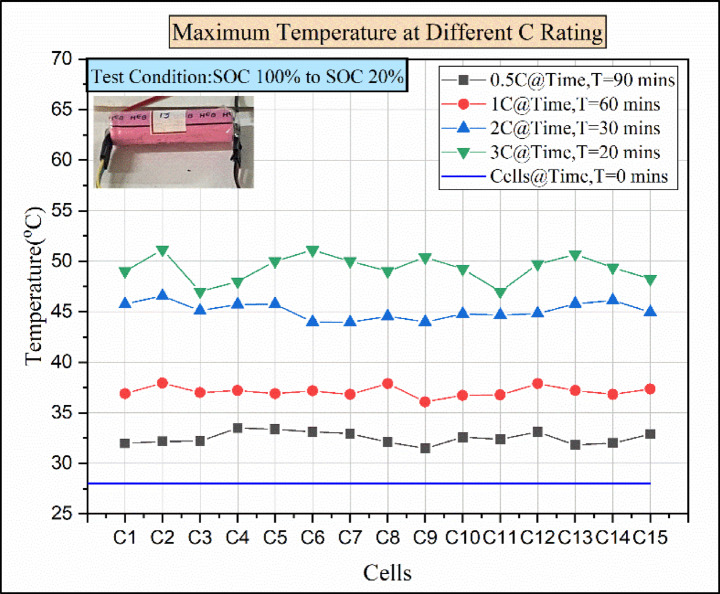
Maximum temperature for various discharge rates for natural convection.

Initially, the experiment was performed to explore the temperature distribution at natural convection conditions. Figure 7 explains the temperature vs. number of cells for different discharging load conditions in natural convection. The density of air is 1.225 kg/m^3^, and the specific heat capacity of air is approximately 1 kJ/kg. The 15 batteries are discharged in the presence of atmospheric air at different load conditions under natural convection conditions. In the 0.5 °C discharging condition, the elevated temperature reached by cell C4 was 33.49 °C. Additionally, cells C5 and C12 show maximum temperatures of 33.38 °C and 33.12 °C. However, the discharging condition rose from 0.5 C to 1 C, and the temperature of the cells C2, C8, and C12 rose above 37.8 °C, while the remaining cells’ temperature was below 37.8 °C.

For the 2 C discharging condition, the cells C2 and C14 reached a maximum temperature of above 46 °C. Compared to 0.5 °C, 1 C, and 2 °C discharging conditions, the 3 °C discharging condition shows more deviation in temperature value. For the 3 C discharging condition, the cell C2 temperature reaches above 51 °C, which is more than the operating value of the Li-ion battery. Without any cooling medium, the generated heat in the battery was released to the surroundings. There were small gaps between the battery modules, which caused an accumulation of heat between the battery cells. Due to a rise in battery temperature, the middle battery is impacted by both the heat produced by the closed battery and the heat produced by the battery itself^27^. For this reason, cell 2 shows the maximum temperature compared to other cells in 1 C, 2 C, and 3 C discharge conditions. For a higher discharging rate, the heat generation rate will be high, and it follows the order of 3 C > 2 C > 1 C > 0.5 C^41^. For every discharging condition, the time taken for discharging is presented in Fig. 7. The batteries discharging from 100 SOC% to 20 SOC% take a total of 90 min from the start to the end at 0.5 C discharging load condition. Similarly, for a 1 C discharging load, the time required for discharging from 100% SOC to 20% SOC would be 60 min. In the 2 C discharging load condition, the time required was 30 min for discharging from 100% SOC to 20% SOC. Similarly, at 3 C discharging load condition, the time required for discharging would be 20 min for discharging the batteries from 100% SOC to 20% SOC. Finally, the result shows that the time taken for discharging condition decreases as the C-rate increases and follows the order of 0.5 C > 1 C > 2 C > 3 C.

**Fig. 8:**
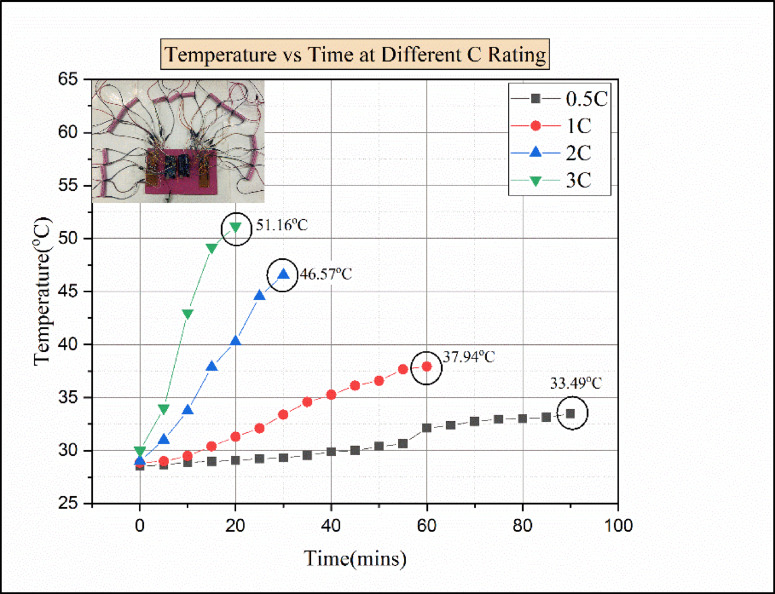
Temperature vs. time for various C ratings for natural convection.

Figure 8 indicates the temperature vs. time at various discharging load conditions in natural convection. The temperature of each cell increases over time in all discharging load conditions. In this experimental analysis, for a 0.5 C discharging load condition, the maximum temperature attained was 33.49 °C. But in 1 C discharging load condition, the maximum temperature rises to 37.94 °C, which is comparatively higher than the temperature of other cells. Due to the increase of internal ohmic heat and poor dissipation of heat to the surroundings, the cell temperature was rising^41^. In the 2 C discharging load condition, the maximum temperature reached was 46.57 °C. Comparatively, the temperature difference between 1 C and 2 C was 8.63 °C, which shows sudden rises in temperature for 2 C discharging conditions. This sudden rise in the temperature of the battery at 2 C discharging load condition was due to the accumulation of heat in the battery, as there was no battery thermal management system^27^. At 3 C discharging load condition, cell C2 attained a temperature of 51.16 °C, which is the highest temperature value among the other cells. The temperature of the battery rises as the load condition increases, and due to that, the capacity of the battery decreases, which leads to degradation of battery performance^27^.

### Temperature distributions for different C rates for the pure PCM battery module

**Fig. 9:**
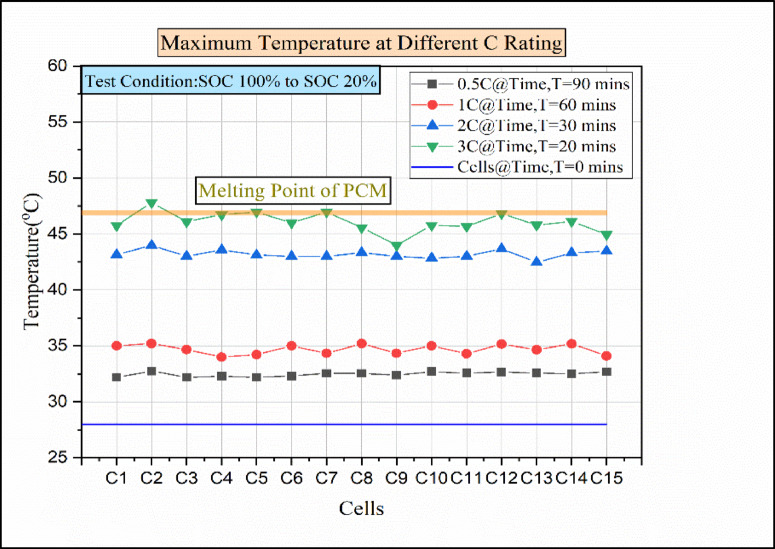
Maximum temperature graph for various discharge rates for pure PCM.

The maximum temperature rise during natural convection for maximum discharge was 51.16 °C. Moreover, the temperature attained was above the operating limit. To manage the temperature, a Passive battery thermal management-only paraffin wax was employed, and the result was analysed. The relationship between cells vs. temperature for the various discharging conditions is represented in Fig. 9. The main purpose of utilising the paraffin wax was its nominal thermal conductivity, high ability to absorb heat, and high latent heat performance. At SOC 100% for time (T = 0 min), the temperature of the cells was around 28 °C. However, cell 2 attained the maximum temperature during the 0.5 °C discharge rate. Other than cell-12, the remaining cells show relatively close temperature values between 32.2 °C and 32.5 °C. When the load condition rises from 0.5 C to 1 C, the time taken for discharging decreases from 90 min to 60 min. For 1 C, there was a fluctuation in each cell temperature. As a result of 1 C, cells 2, 6, 8, 10, 12, and 14 obtained a maximum temperature above 35 °C. Meanwhile, the remaining cells show a temperature value below 35 °C.

**Fig. 10:**
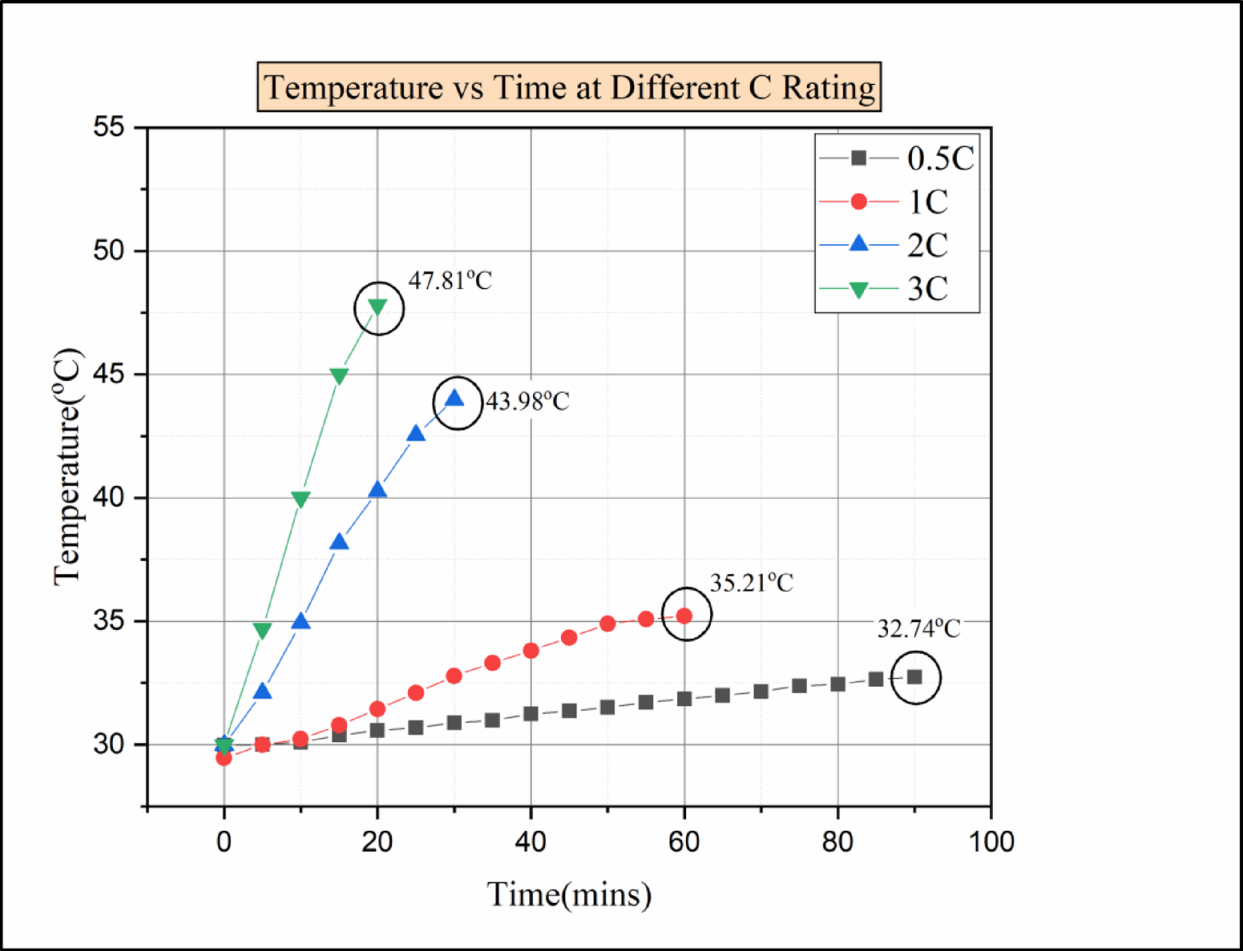
Temperature vs. time for various C rating rates for pure PCM.

The reason behind the fluctuation in cell temperature is due to the lower heat dissipation rate in paraffin wax; moreover 49, it has low thermal conductivity^42^, which leads to rises in temperature on the battery surface^43^. Consequently, when the discharging condition was raised from 1 C to 2 C, there was a sudden rise in the temperature of the cell. Compared to 1 C, the cell temperature increases above 43 °C in 2 C conditions, which shows a temperature difference of 8 °C. There was a huge temperature difference between 1 °C and 2 °C conditions. The main reason was the decrease in thermal conductivity as the temperature of the cell was close to the melting point of the paraffin wax (melting point-46 °C)^44^. The temperature of the cell for the 3 C discharge condition was even higher than the paraffin wax’s melting point. The greatest temperature reached was recorded by Cell 2 as 47.81 °C, which was above the melting point of paraffin wax.

From Fig. 10, the time vs. temperature for various C ratings under the condition of the passive medium (paraffin wax only) is presented. We can identify that the temperature increases over time in all discharging load conditions. From SOC 100% to SOC 20%, the maximum temperature for every minute was noted. Moreover, the maximum temperature attained during the 0.5 °C discharge condition was 32.74 °C. However, for the 1 C load condition, the maximum temperature obtained was 35.21 °C. Furthermore, when the load condition increases from 1 C to 2 C temperature rises to 43.98 °C. The temperature increased because the latent heating phase time decreased, and as Anuj Kumar et al. reported, the temperature reached the paraffin wax melting point^44^. Moreover, the maximum temperature attained was 47.81 °C for higher discharge conditions of 3 C rate. The increase in temperature was due to the continuity of the post-melting sensible heating phase after the completion of the latent heating phase^29^. For the 3 C discharging condition, the temperature reaches the melting point of paraffin wax. The temperature attained during the 3 C discharge condition was not within the operating limit of the battery. So, some enhancement has to be made to attain the operating limit of the battery.

### Investigations of PCM with additives

#### Investigation of paraffin wax with 5% Al_2_O_3_

**Fig. 11:**
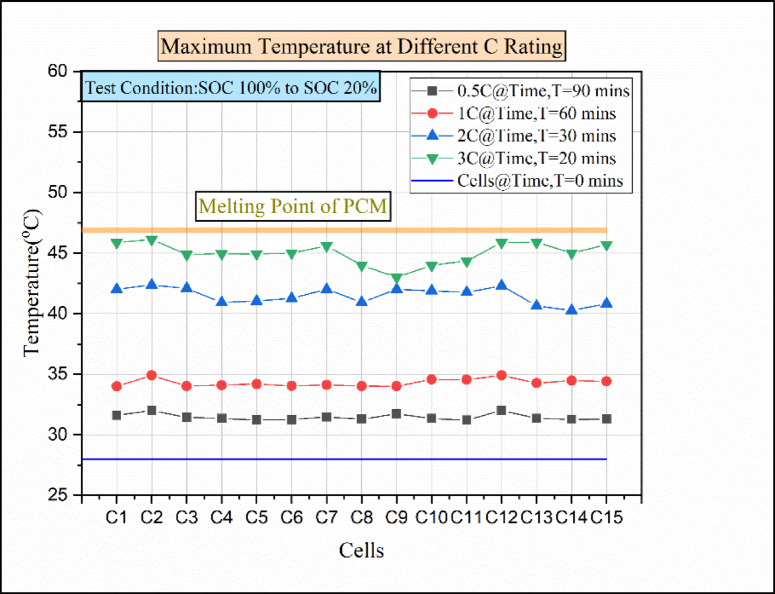
Maximum temperatures for various discharge rates of paraffin wax with 5% Al_2_O_3_.

According to the PCM analysis, an ineffective battery thermal management system may result when the cell temperature climbs above the paraffin wax’s melting point, which lowers latent heat and the rate at which heat is absorbed. That is why to enhance the paraffin wax property, Al_2_O_3_ nanoparticles were added in this experiment. Figure 11 indicates the temperature vs. cells at various discharging load conditions in the passive medium (paraffin wax with 5%Al_2_O_3_). As a result, the cells C2 and C12 attained the maximum temperature of 32 °C in 0.5 °C discharging conditions. In the 1 C discharging condition, the maximum temperature attained by the cells was above 34 °C. In addition to that, the cells at 1 C discharge condition almost attained equal temperature.

The reason behind the uniform rise in cell temperature was due to the rise in heat absorption rate in PCM with the addition of nanoparticles^9^. While in 2 C discharging load conditions, the cells C1, C2, C3, C7, C9, and C12 reached a higher temperature of above 42 °C. At the 3 C discharging load condition, the maximum temperature of the cells reached above 46 °C, which is the maximum value of temperature concerning other cells. The cells that attained the maximum temperature value in 3 C discharging load conditions are not the same. Due to the limited conduction heat transfer rate between the nano-enhanced paraffin wax and cells, the temperature of these cells was higher than that of other cells^26^.

**Fig. 12:**
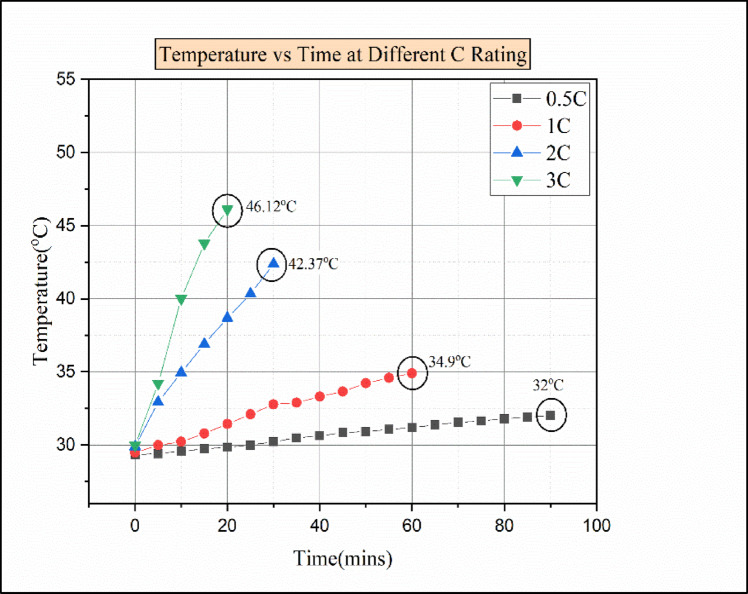
Temperature vs. time graph for various C ratings of paraffin wax with 5% Al_2_O_3_.

The Fig. 12 indicates the temperature vs. time for various C ratings. From the result, it is identified that the temperature increases over time in all discharging load conditions. Because of the specific heat capacity of paraffin wax (2.3–2.9 kJ/kg. K) and the low thermal conductivity of paraffin wax (0.2 W/m K), the addition of 5% of Al2O3 Nanoparticle to the paraffin wax, the thermal conductivity of the passive medium would be sufficiently increased.

This passive BTMS medium can decrease the battery module temperature as much as possible. At a 0.5 °C discharging rate, the battery attains the maximum temperature of 32 °C after 90 min from the start of discharge. Similarly, at the 1 C discharging load conduction, the battery attains the maximum temperature of 34.9 °C at the time 60 min from the start of discharge. In the 2 C discharging load condition, the battery attained the maximum temperature of 42.37 °C in 30 min from the start of discharge. Moreover, from the 1 C to 2 C discharging condition, there was a sudden rise in cell temperature from 34.9 °C to 42.37 °C, with a temperature difference of 7.47 °C. This was due to the low dissipation of heat to nano-enhanced PCM^9,32^. In the 3 C discharging load condition, the batteries attained the maximum temperature of 46.12 °C in 20 min from the start of discharge. However, the maximum temperature attained during the 3 C discharge rate was below the melting point of the paraffin wax. The PCM thermal conductivity increases with the addition of nanoparticles. In addition to that, the heat transfer rate increases, and it also changes the viscosity, density, and PCM’s latent heat capacity^26,33^.

#### Investigation of paraffin wax with 10% Al_2_O_3_

**Fig. 13:**
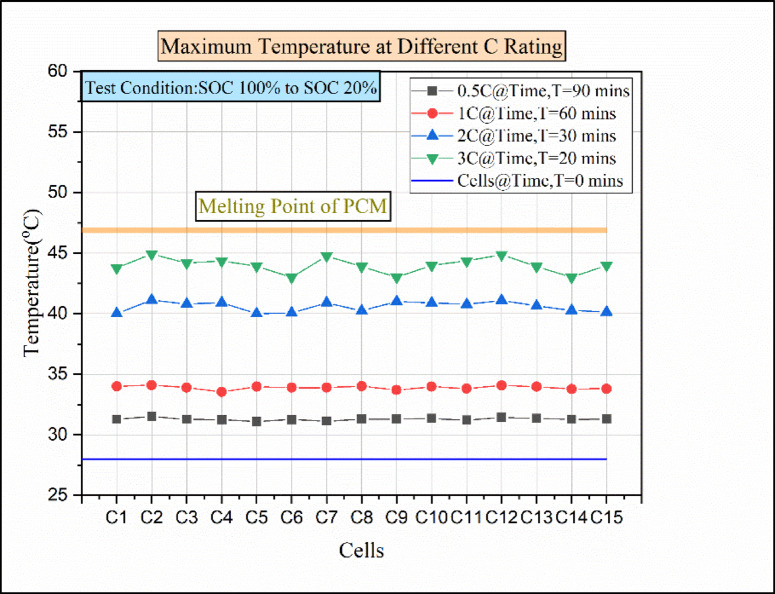
Maximum temperatures for various discharge rates of paraffin wax with 10% Al_2_O_3_.

The thermal conductivity of Al_2_O_3_ nanoparticles (20–30 W/mK) and the melting point of Al_2_O_3_ nanoparticles (2041 °C) will help to improve the withstanding capacity of the passive medium. Figure 13 indicates cells vs. temperature at various discharging load conditions in the passive medium (paraffin wax with 10% Al_2_O_3_). The study was initiated in steady-state conditions. At the initial time, t = 0 min, the temperature of the cells was almost equal to the atmospheric temperature value. For the 0.5 C discharge load condition, the maximum temperature reached by all the cells was above 31 °C. Moreover, all the cells attained equal rises in temperature, which was due to the equal dissipation of heat all over the passive medium. Similarly, in the 1 C discharge condition, the maximum temperature reached above 33.5 °C in each cell. Similar to the 0.5 °C discharge condition, the 1 C discharge condition shows an equal rise in cell temperature. The addition of Al nanoparticles with high-conductive characteristics increases the thermal conductivity of the PCMs^26,34^. In addition to that, the heat transfer capacity is enhanced in the composite PCMs. Similar to the above statement addition of nanoparticles increased the thermal conductivity of the PCM due to that the heat transfer capacity of the PCM increased. For this reason, the cells show an equal rise in temperature at the 1 °C discharge condition. Then, for a higher discharge condition of 2 C, the maximum temperature attained by each cell was above 40 °C. Finally, for the 3 C discharge condition, the maximum temperature attained was above 44.5 °C by the cells C2, C7, and C12. However, the remaining cells show a temperature value below 44.5 °C This was due to the addition of nanoparticles from 5 to 10%, which enhances the thermal storage performance of the paraffin wax^31,35^.

**Fig. 14:**
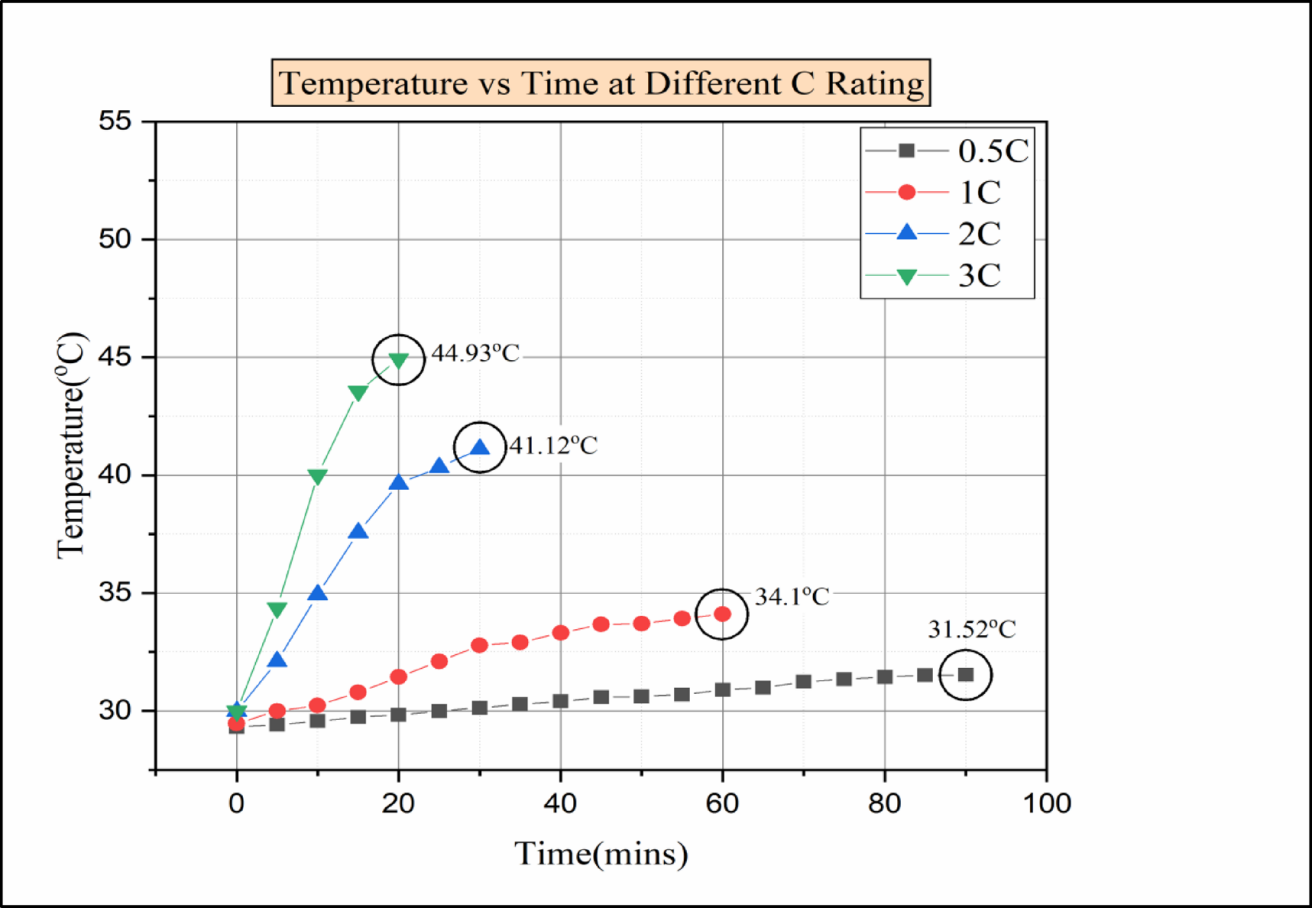
Temperature vs. time for various C ratings of paraffin wax with 10% Al_2_O_3_.

The Fig. 14 indicates the time vs. temperature for various C ratings. However, the addition of nanoparticles from 5 to 10% increases the heat transfer ability of nano composite-based PCM when compared to the pure PCM, as explained by Anuj Kumar et al.^44^. In this experimental investigation (paraffin wax with 10% Al_2_O_3_), for the 0.5 °C discharge condition, the maximum temperature attained was 31.52 °C. Similarly, the maximum temperature of 34.1 °C was reached during the 1 C discharge condition. Moreover, at higher C-rating, i.e., for 2 C and 3 C discharger conditions, the maximum temperature attained was 41.12 °C and 44.93 °C, respectively. The melting point of the pure PCM was not affected by the addition of nanoparticles with the pure PCM. Furthermore, the latent heat of fusion of PCM is lowered by the addition of nanoparticles^44^. Ultimately, the reduction in latent heat of fusion causes the thermal conductivity of nano-enhanced PCM to rise. In conclusion, the addition of nanoparticles at 5–10% helped to reduce the rise in temperature of the cells, which was due to the increase in thermal conductivity of the nano-enhanced PCM. Even though the maximum temperature attained during the 3 C discharge condition was not within the operating limit of the Li-ion battery.

#### Investigation of paraffin wax with 15% Al_2_O_3_

**Fig. 15:**
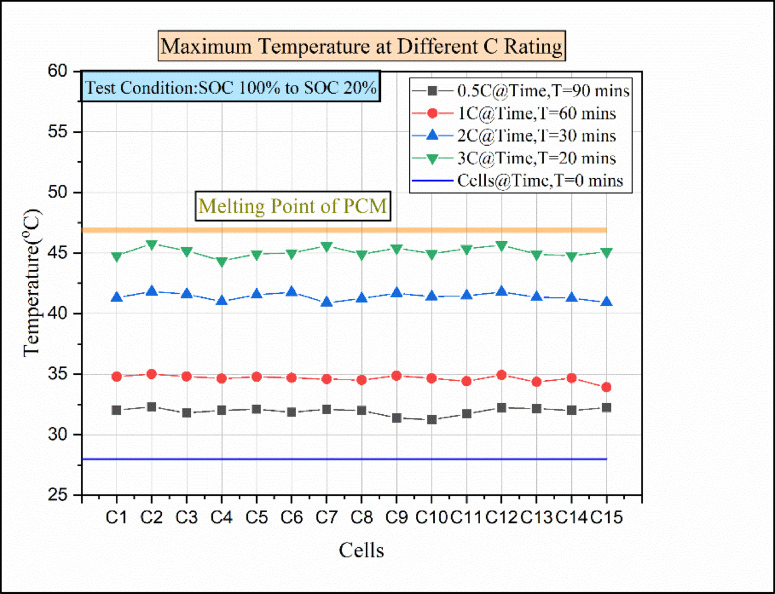
Maximum temperatures for various discharge rates of paraffin wax with 15% Al_2_O_3_.

Figure 15 indicates the cells vs. temperature at various discharging load conditions in the passive medium (paraffin wax with 15% Al_2_O_3_). In the 0.5 °C discharging condition, the cell’s temperature rises above 32 °C. The cells then reached a maximum temperature of above 34 °C under the 1 C discharging condition. However, the cells C2 and C12 reached the maximum temperature in both discharging conditions. The reason behind in rise of the cell temperature was due to the addition of nanoparticles at a higher concentration which decreased the amount of pure PCM so that during the phase change process less amount energy was absorbed by nano-enhanced PCM compared to pure PCM as described by Anuj Kumar et al. (2021)^30^. For the 2 C discharging condition, the cells reached a maximum temperature of above 41 °C. Similarly, in the 3 C discharging condition, the cells attained a maximum temperature of above 44 °C. The increase in dosage level content results in varying the heat-conducting performance. Similarly, the addition of Al_2_O_3_ at a higher concentration to the PCM results in the low heat-conducting performance of nano-enhanced PCM. This was the reason for the rise in cell temperature during 2 C and 3 C discharging conditions^26,36^.

**Fig. 16:**
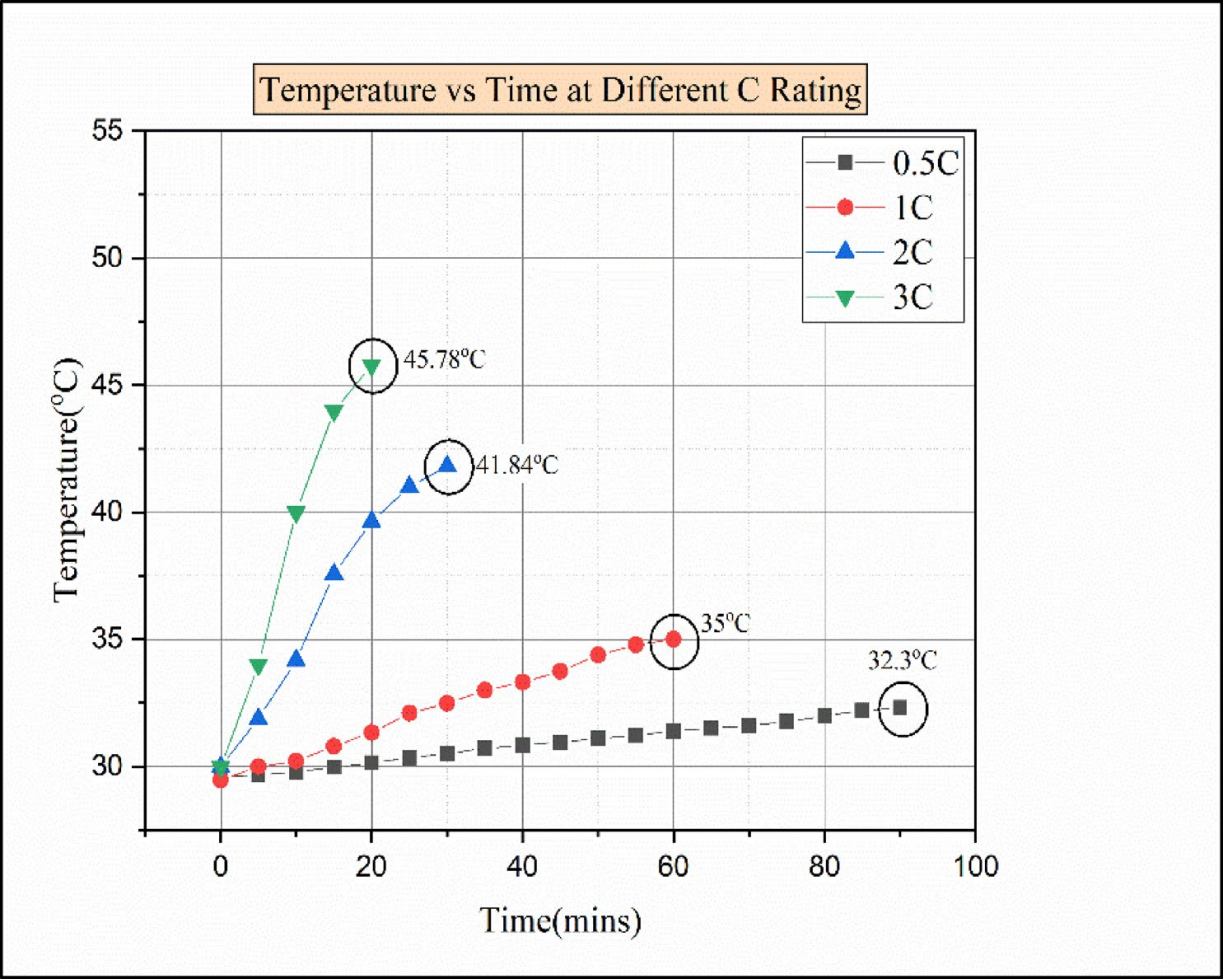
Temperature vs. time for various C ratings of paraffin wax with 15% Al_2_O_3_.

Due to an increase in discharging load conditions, the time taken for discharging decreases, and meanwhile, the temperature increases for higher C-rates. Figure 16 indicates the temperature vs. time for various C ratings. At a 0.5 °C discharging rate, after 90 min, the cell reached the elevated temperature of 32.3 °C. Similarly, at the 1 C discharging load conduction, the cell attains a higher temperature of 35 °C at the time 60 min from the start of discharge. Moreover, the cell attained the elevated temperature of 41.82 °C in 30 min from the start of discharging in a 2 C discharging load condition. In the 3 C discharging load condition, the batteries reached the maximum temperature of 45.78 °C in 20 min from the start of discharge. Compared to 10% of Al_2_O_3_, 15% of Al_2_O_3_ results show a slight rise in temperature during the discharge condition. The reason for the temperature rise was due to the excess addition of Al_2_O_3_ in PCM and the improper combination. This drawback results in sedimentation and an increase in thermal resistance, so that the thermal storage performance is reduced in nano-enhanced PCM^29^. Moreover, the economic benefits of PCM even diminish because of larger quantities of Al_2_O_3_ nanoparticles present in the PCM^29^. So, 10% of Al_2_O_3_ nanoparticles with paraffin wax would be an optimal choice in hybrid battery thermal management.

#### Investigation of hybrid BTMS

**Fig. 17:**
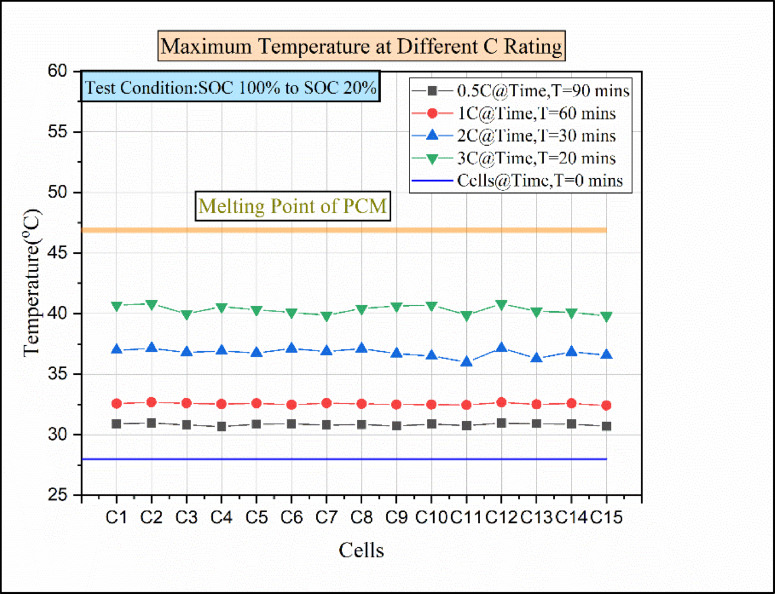
Maximum temperatures for various discharge rates for hybrid BTMS.

From the previous experimental investigation, the maximum temperature attained was above 40 °C for the 3 C discharge condition. To maintain the temperature of the cell below 40 °C and to maintain the cells within the operating limit, a hybrid battery thermal management was employed. Because of the presence of circulating water and the nano-enhanced PCM medium, the temperature of the cell can be reduced further. Figure 17 indicates the cells vs. temperature at the various discharging conditions in the hybrid battery thermal management. In the 0.5 °C discharging condition, the temperature rise in the cells was almost equal. The cells attained a maximum temperature of above 30.5 °C. Similar to 0.5 °C, the rise in cell temperature was almost equal in the 1 °C discharging condition. The maximum temperature attained by the cells was above 32 °C. The equal temperature rise in the cell was due to the uniform heat dissipation by the nano-enhanced PCM and high heat conduction by the cooling liquid. In the 2 C discharging condition, the cells reached a maximum temperature of above 36 °C. Similarly, for the 3 C discharging condition, the maximum temperature reached by the cells was above 39 °C. Moreover, for 2 C and 3 C discharging conditions, cell C12 shows a maximum temperature higher than the other cells. In water circulation through the copper coil, the initial cells cool first so that the final cells become warmer^44,34^. This was the reason for the rise in temperature for cell C12 in 2 C and 3 C discharging conditions.

**Fig. 18:**
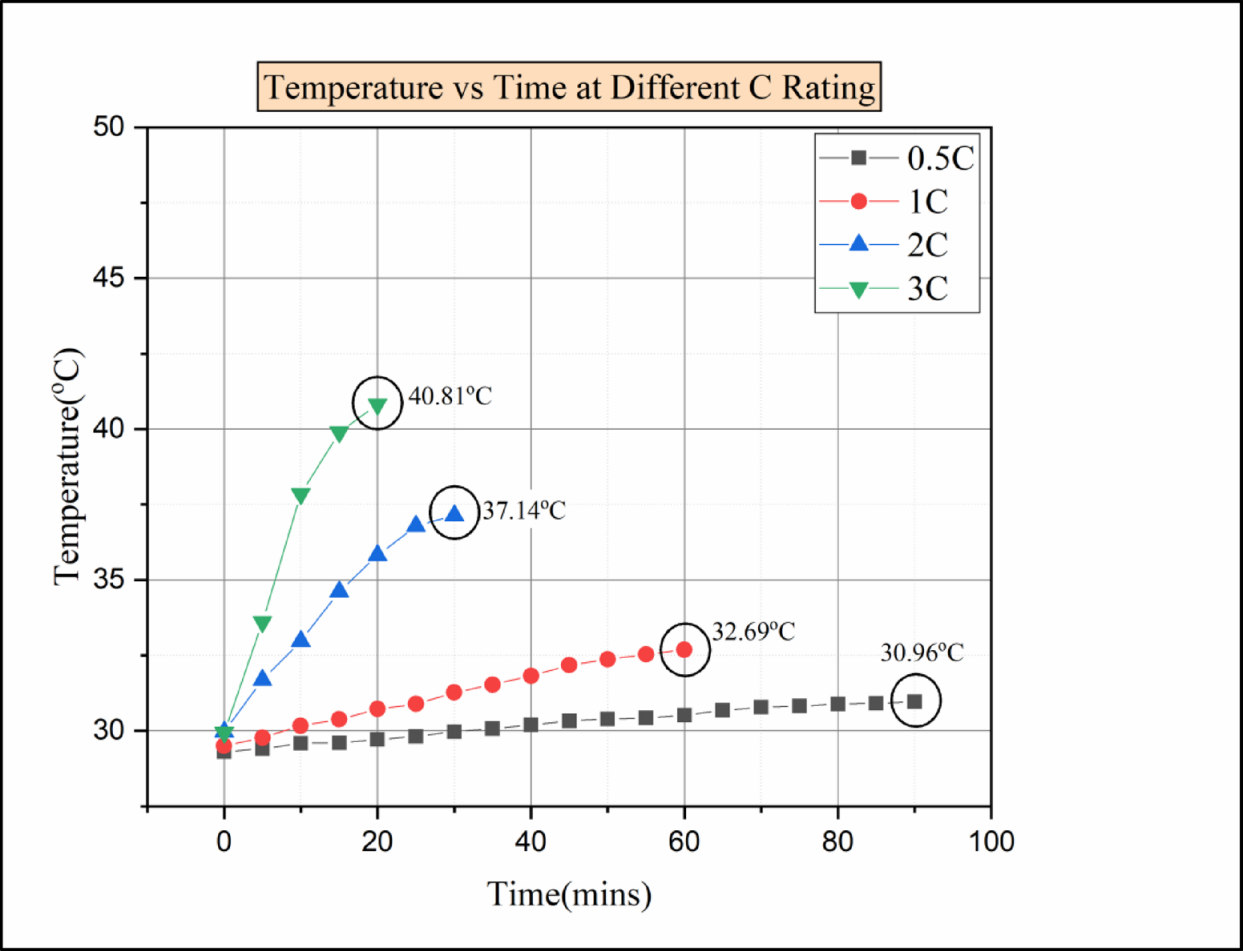
Temperature vs. time for various C ratings for Hybrid BTMS.

Figure 18 represents the time vs. temperature for various C ratings. The thermal performance of hybrid batteries is primarily determined by the PCM’s thermal conductivity, the coolant’s mass flow rate, and the coolant’s flow direction. The temperature rise in the cell may depend on the inlet coolant temperature. The cooler’s increased input temperature causes the temperature differential between the battery pack and the coolant to diminish, making it harder for the heat produced by the battery to dissipate. These result in a decreased driving force for heat transfer^39^. So that the inlet coolant temperature was maintained within 27 °C. In the 0.5 °C discharging condition, the maximum temperature reached by the cell was 30.96 °C. Then, for the 1 C discharging condition, the maximum temperature attained by the cell was 32.69 °C. For 2 C and 3 C discharging conditions, the maximum temperature obtained was 37.14 °C and 40.81 °C, respectively. Moreover, the maximum temperature reached was comparatively lower than other battery thermal management systems. This was mainly due to the cooling liquid because the heat absorbed by the PCM was easily taken away by the cooling liquid, so the thermal accumulation was prevented in the system^25^. For this reason, the temperature rise in the cell for 3 C was within 40 °C. The novel hybrid cooling system helps in balancing the cooling efficiency between the liquid cooling system and the PCM. It also provides a safe operating condition for Li-ion batteries^39,34^.

### Overall comparison of various cooling methods for battery modules

**Fig. 19:**
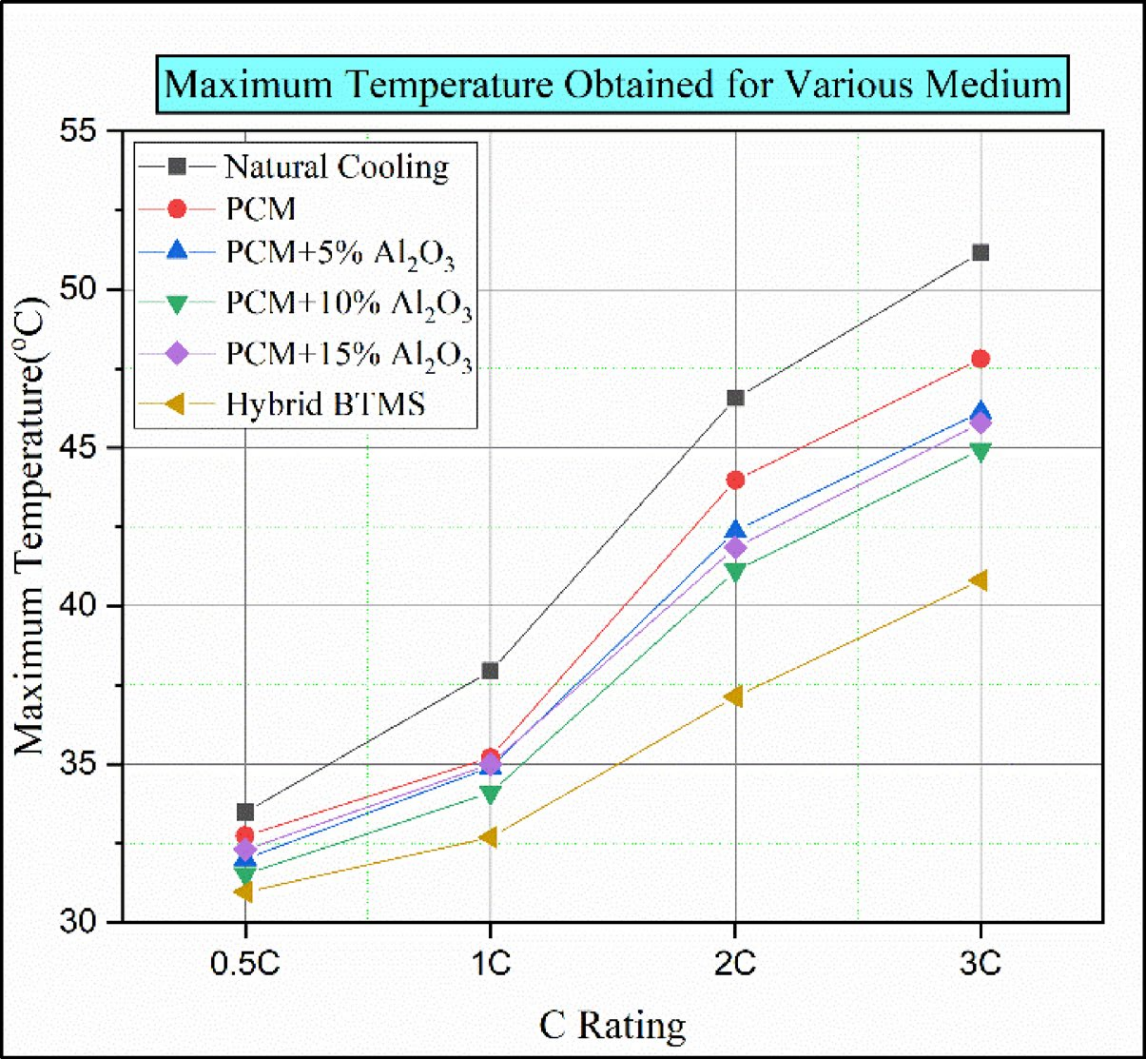
Maximum temperature for various discharge rates at different cooling medium.

Figure 19 shows the maximum temperature versus C rating for various conditions, such as natural convection, using only PCM (pure paraffin), PCM enhanced with nanoparticle additives (PCM with 5% Al2O3, 10% Al_2_O_3_, 15% Al_2_O_3_), and a hybrid model (containing both active and passive elements). Furthermore, in natural convection, the batteries’ temperature is higher. Figure 19 indicates that the battery’s highest temperature in natural convection at a 3 °C discharge rate was 51.16 °C. In the case of PCM-only (pure paraffin), the maximum temperature rise was 47.81 °C at the highest discharge rate of 3 C. Due to the heat dissipation properties and the high latent heat of paraffin wax, the battery temperature is lower compared to that in natural convection^37^. Phase change material augmented with 5% Al_2_O_3_ allows the battery to reach a maximum temperature of 46.12 °C under 3 C discharge conditions. The reduction in the temperature of the battery in this case was due to the increased heat-carrying capacity of the PCM medium resulting from the addition of 5% Al2O3 to paraffin wax^29^. By using paraffin wax enhanced with 10% Al_2_O_3_ as a passive medium, the battery temperature further decreased to 44.93 °C at a 3 C discharging condition. The addition of Al_2_O_3_ nanoparticles in a higher ratio increases the overall thermal conductivity of the passive medium. However, in the case of paraffin wax enhanced with 15% Al_2_O_3_ nanoparticles, the batteries’ temperature was slightly higher than in the previous case (paraffin wax with 10% Al_2_O_3_ medium).

While adding Al_2_O_3_ nanoparticles at a higher concentration, agglomeration of nanoparticles can occur in the passive medium, creating a barrier to heat transfer. Consequently, the battery temperature would increase in this scenario. In hybrid battery thermal management, the active system (coolant water at a flow rate of 400 L/hour) would absorb heat, and the optimal passive medium (paraffin wax with 10% Al_2_O_3_) was used in this hybrid model. Therefore, the maximum battery temperature under a 3 C discharge load condition in this hybrid BTMS was 40.81 °C. By implementing this hybrid BTMS, we can lower the battery temperature by around 10 °C.

From the above observations, the selection of paraffin wax as a phase change material (PCM) is despite its relatively high melting point of 40–60 °C. Paraffin wax was chosen for its high latent heat capacity of 250 kJ/kg, chemical stability^45^, non-corrosive nature^46^, cost-effectiveness^47^, and ease of integration with nanomaterials, as reported in previous studies^38,48^. Although the melting point of 57–62 °C of the nano-enhanced PCM is above the ideal operating range of lithium-ion batteries, typically 25–45 °C, the PCM in this study was strategically intended to serve as a thermal buffer in emergency or extreme 3 C discharge load conditions, rather than during normal operation. This approach aligns with findings in the literature^49^, where PCM with melting points between 55 and 65 °C was used to delay runaway temperature rise during high discharge rates or cooling system failures. While full melting was not observed at lower discharge rates (0.5 C, 1 C), signs of partial melting and thermal plateau behavior were noted at 2 C and 3 C discharge conditions. This suggests that the latent heat absorption mechanism of the nano-doped PCM was partially utilized in high-stress scenarios.

### Computational analysis

**Fig. 20:**
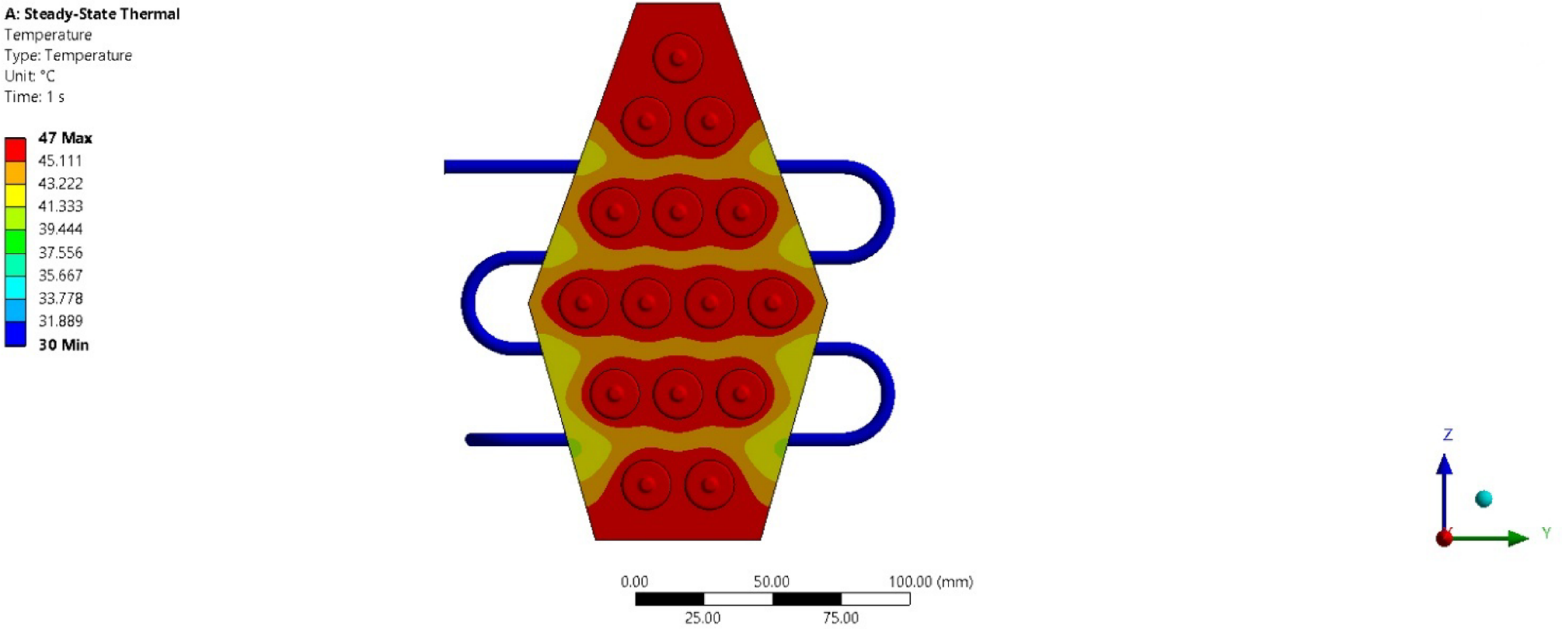
Temperature contour plot for pure PCM battery module.

**Fig. 21:**
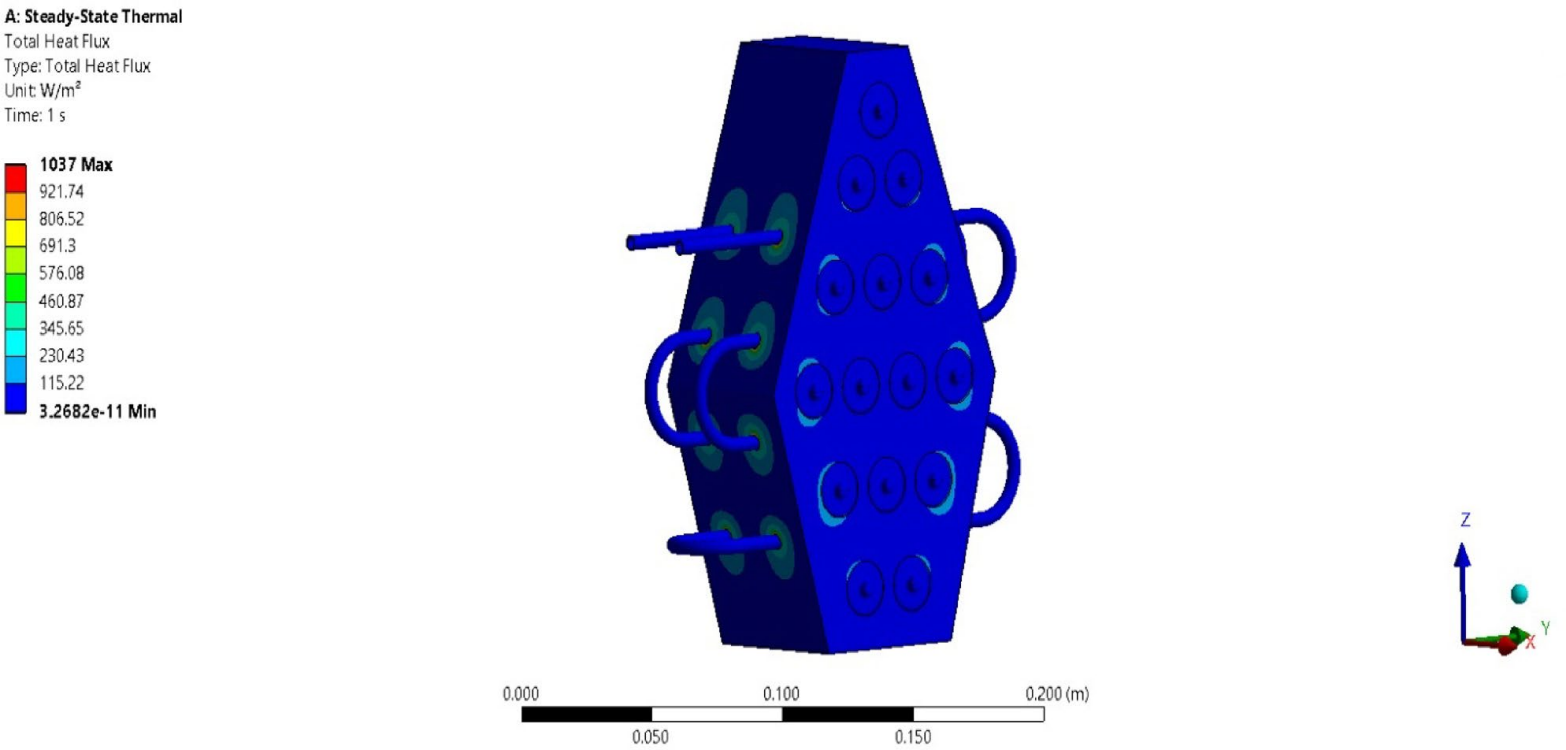
Heat transfer rate for pure PCM battery module.

**Fig. 22:**
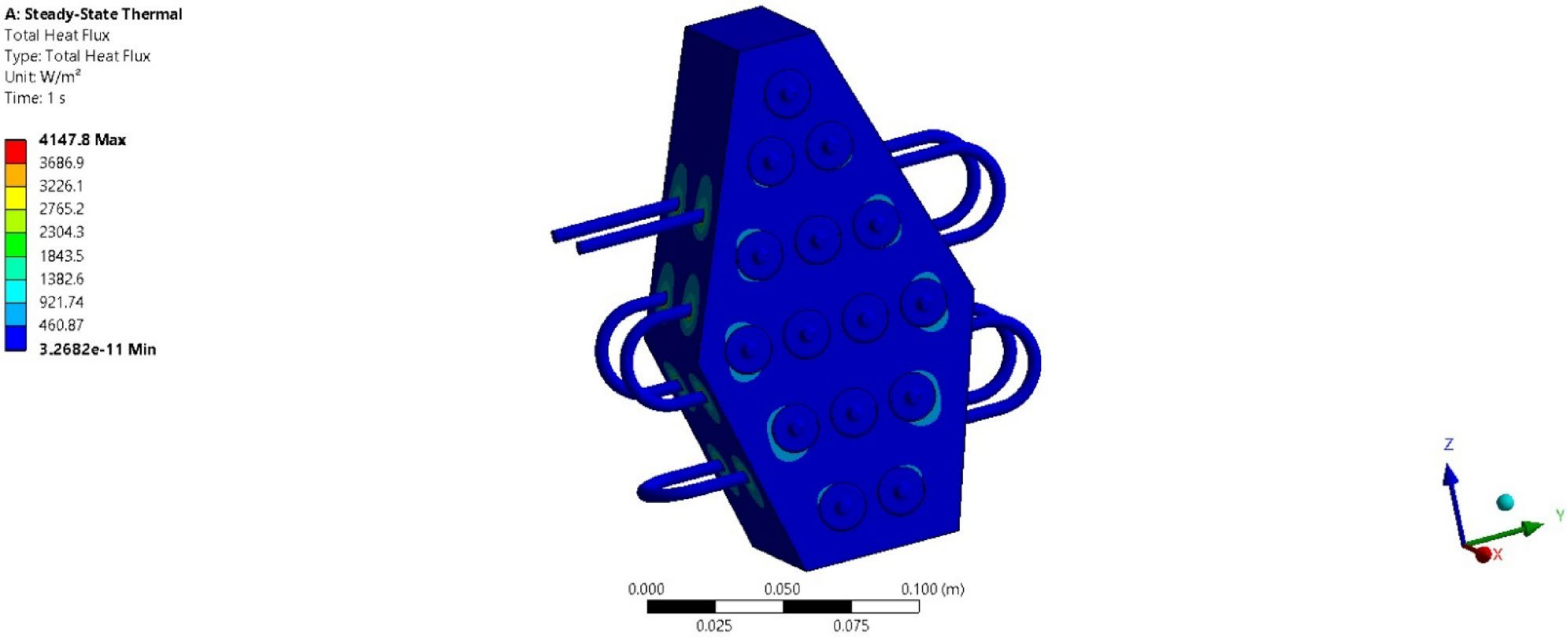
Heat transfer rate for PCM + 15% Al_2_O_3_ battery module.

To validate the experimental results, a numerical study is conducted under extreme operating conditions (3 C discharge rate) for pure paraffin and its PCM + Al_2_O_3_ combinations. Figure 20 shows a temperature contour plot while the battery module operates at 3 C discharge conditions. The resulting plot indicates a maximum temperature of about 47 °C, and the average temperature is computed as 46.34 °C, which aligns well with the experimental results. The error percentage obtained for this condition is 1.4%. The maximum temperature is observed near the batteries and decreases upon reaching the PCM surface. This demonstrates that heat transfer occurs from the batteries to the PCM slowly and steadily. Further studies have been conducted to evaluate the heat transfer rate for pure PCM and its various composite combinations. Figure 21 presents the heat transfer rate plot for pure PCM, while Fig. 22 presents the heat transfer rate for PCM + 15% Al_2_O_3_. The total heat flux transferred in all three directions is consolidated and shown in Table 4.

**Table 4:** Total heat flux rates.

Description	PCM	5%	10%	15%
Total heat flux (W/m^2^)	1037	2073.9	3214.6	4147.8
X-heat flux (W/m^2^)	830.6	1661.2	2574.9	3322.4
Y-heat flux (W/m^2^)	264.4	528.8	819.63	1057.6
Z-heat flux (W/m^2^)	983.19	1966.4	3047.9	3932.7

For the pure PCM battery module, a total heat flux of about 1037 W/m^2^ has been obtained, with maximum heat transfer occurring in the Z direction. Similarly, as indicated in Table 4, total heat flux values of approximately 2073, 3214, and 4147 W/m^2^ are noted for the pure + 5% Al_2_O_3_ combinations. In all cases, the maximum heat flux rate is obtained along the Z direction, and the total heat flux increases as the Al_2_O_3_ percentage rises.

The main reason behind this scenario is that as the concentration of Al_2_O_3_ rises, thermal conductivity also increases. These nanoparticles act as heat sinks and effectively carry away the heat generated by batteries in all directions^46,47^.

The computational analyses have proved the reliability of the imposed experimental procedure and its obtained outcomes.

## Conclusion

In this study, a hybrid-based BTMS is implemented for the lithium-ion battery module, and a detailed experimental study was carried out. Experiments were carried out at free convection, pure paraffin, PCM with Al_2_O_3_ (5%, 10%, and 15%), and hybrid (both active and passive). Table 5 compares the present study in comparison with the previous study.

**Table 5:** Comparison of the present study with the previous study.

BTMS method	Peak temperature at 3 C	Observation	References
Natural convection	51.16 °C,	Improved conductivity, Partial phase change	Current study
Pure PCM (paraffin only) Nano-Enhanced PCM (10% Al_2_O_3_),	47.81 °C,		
Hybrid BTMS	44.80 °C		
(PCM + Counterflow water cooling)	40.81 °C		
Air cooling (Fan-based)	55–60 °C	Limited by the low heat capacity of air	^2^
PCM-only (paraffin)	48–50 °C	Effective up to 1–2 C only	^25^
Liquid cooling (water)	42–44 °C	High thermal control but energy-intensive	^26^
Advanced nanofluid-based cooling	39–41 °C	Require pump power and nanofluid management	^25^

From the above observation, this study summarises that.

1. The hexagonal battery module layout also enhanced thermal uniformity across cells by minimising heat accumulation at the core and improving heat dissipation pathways, which support more stable and efficient operation under high C-rate.
2. Under natural convection, the battery reached a peak temperature of 51.16 °C at 3 C, which exceeded the safe operational threshold. However, when pure paraffin wax was used, the temperature decreased to 47.81 °C, indicating an improvement of approximately 3.35 °C.
3. To address the low thermal conductivity of paraffin, Al_2_O_3_ nanoparticles were incorporated into the PCM in varying concentrations. Among the tested formulations, the 10% Al_2_O_3_ nano-doped PCM demonstrated the best thermal performance, with effective heat absorption and uniform dispersion, as confirmed by SEM and FTIR analysis.
4. In the case of hybrid BTMS, combining 10% Al_2_O_3_-enhanced PCM with active water cooling, the maximum temperature attained is 40.81 °C at a 3 C condition. When compared to natural convection, the temperature was reduced by around 10.35 °C by using this hybrid battery thermal management system. This configuration successfully maintained the battery within its recommended operating range, enhancing both the thermal safety and system reliability of a Li-ion battery.
5. A simulation attempt is conducted by running the battery at a 3 C discharge rate for the Pure PCM battery module. The computed average temperature was measured at 46.34 °C, which shows excellent agreement with the experimental results. The corresponding error percentage for this condition was calculated to be 1.4%, indicating a high level of accuracy in the simulation. Similarly, the total heat flux increases as the percentage of Al_2_O_3_ in the paraffin matrix rises.
6. While Al_2_O_3_ is advantageous due to its affordability, chemical stability, and thermal conductivity, effectively scaling up the use of nano-enhanced PCM can be achieved by using stabilizers or surface-functionalized nanoparticles to improve dispersion stability. Although higher nanoparticle concentrations can enhance thermal conductivity, they also result in increased material and processing costs, which may not be justifiable for large-scale BTMS deployment. Practical limitations such as handling difficulties, increased viscosity, and reduced thermal performance gains from agglomeration further support the choice of 10 wt% Al_2_O_3_ as an optimal trade-off between effectiveness and economic viability.

## Future work

Future work will focus on overcoming nanoparticle agglomeration at higher loadings, employing techniques such as surface treatment of Al_2_O_3_ nanoparticles, incorporation of dispersing agents, and optimization of mixing protocols to maintain uniform dispersion and maximize thermal performance.

## Data Availability

Data availabilityThe datasets used and/or analysed during the current study available from the corresponding author on reasonable request.

## Abbreviations

BTMS: Battery thermal management system
PCM: Phase change material
Al_2_O_3_: Aluminium oxide
Li-ion: Lithium-ion
EV: Electric vehicle
SOC: State of charge
CuO: Copper oxide
TMS: Thermal management system
K: Kelvin
LC: Liquid cooling
LPH: Litres per hour
Pure PCM: 100% phase change material
PCM + 5% Al_2_O_3_: 95% phase change material + 5% aluminium oxide nanoparticle
PCM + 10% Al_2_O_3_: 90% phase change material + 10% aluminium oxide nanoparticle
PCM + 15% Al_2_O_3_: 85% phase change material + 15% aluminium oxide nanoparticle
V: Voltage
DC: Direct current
AC: Alternating current
EG: Expanded graphite
LC: Liquid cooling
BMS: Battery management system
